# National Assessment of Human Health Effects of Climate Change in Portugal: Approach and Key Findings

**DOI:** 10.1289/ehp.8431

**Published:** 2006-07-11

**Authors:** Elsa Casimiro, Jose Calheiros, Filipe Duarte Santos, Sari Kovats

**Affiliations:** 1 Scenarios, Impacts and Adaptation Measures (SIAM) Project, Faculdade de Ciências da Universidade de Lisboa, Lisbon, Portugal; 2 Instituto Dom Luiz, Faculdade de Ciências da Universidade de Lisboa, Lisbon, Portugal; 3 Faculdade de Ciências da Saúde, Universidade da Beira Interior, Covilha, Portugal; 4 Departamento de Engenharia Química, Faculdade da Universidade do Porto, Porto, Portugal; 5 Departamento de Fisica, Faculdade de Ciências da Universidade de Lisboa, Lisbon, Portugal; 6 Department of Public Health and Policy, London School of Hygiene and Tropical Medicine, London, United Kingdom

**Keywords:** climate change, disease, health impact assessment, Portugal

## Abstract

In this study we investigated the potential impact of climate change in Portugal on heat-related mortality, air pollution–related health effects, and selected vectorborne diseases. The assessment used climate scenarios from two regional climate models for a range of future time periods. The annual heat-related death rates in Lisbon may increase from between 5.4 and 6 per 100,000 in 1980–1998 to between 8.5 and 12.1 by the 2020s and to a maximum of 29.5 by the 2050s, if no adaptations occur. The projected warmer and more variable weather may result in better dispersion of nitrogen dioxide levels in winter, whereas the higher temperatures may reduce air quality during the warmer months by increasing tropospheric ozone levels. We estimated the future risk of zoonoses using ecologic scenarios to describe future changes in vectors and parasites. Malaria and schistosomiasis, which are currently not endemic in Portugal, are more sensitive to the introduction of infected vectors than to temperature changes. Higher temperatures may increase the transmission risk of zoonoses that are currently endemic to Portugal, such as leishmaniasis, Lyme disease, and Mediterranean spotted fever.

In this article we describe the Climate Change in Portugal: Scenarios, Impacts and Adaptation Measures (SIAM) project. The first phase of the project was conducted to assess climate change impacts and adaptation measures in continental Portugal (Santos et al. 2002). The SIAM project was divided functionally into 10 groups and an integration team. Seven groups worked on climate change impacts and adaptation measures for specific sectors (impact groups): water resources, coastal zones, agriculture, human health, energy, forests and biodiversity, and fisheries. The remaining groups worked on climate and climate scenarios, socioeconomic scenarios, and a sociologic analysis of climate change issues in Portugal. To facilitate integration across sectors, groups used the same suite of climate data (observed and scenarios) and socioeconomic scenarios.

The results were communicated in Portuguese and in English to the public, decision makers, and other scientists. Throughout the assessment process there were many consultations/interviews with experts (international and national), other stakeholders, and with the other SIAM project groups to discuss cross-sector issues.

In this article we describe the SIAM health impact assessment, focusing on the methods used and the main quantitative results for heat-related mortality, air pollution–related health effects, and vectorborne diseases. Detailed information including suggested adaptation measures for all health impacts assessed is available in the SIAM health technical report (Casimiro and Calheiros 2002).

## Health Impact Assessment Methods

Climate-sensitive health outcomes included in the assessment were identified for Portugal on the basis of previous national and international assessments (McMichael and Githeko 2001). Potential health outcomes identified were heat-related mortality, air pollution–related health effects, vector- and rodent-borne diseases, water- and foodborne diseases, and health effects associated with floods and drought. Table 1 lists health outcomes further described in this article. During the assessment of each health outcome, the following questions were addressed: *a*) What is the current (or historical) burden of the health outcome in Portugal? *b*) What is the climate–health relationship for this health outcome? *c*) Assuming the climate–health relationship to be valid for all exposure scenarios, what climate change health impacts are anticipated for Portugal?

The current burden of climate-sensitive diseases was obtained from national monitoring and control programs as well as from the literature. Where there were sufficient health and climate data, such as for heat-related mortality, epidemiologic analyses were used to identify and quantify relationships between weather variables and health outcomes. In the case of indirect climate change impacts, such as air pollution–related health outcomes and vectorborne diseases, we focused on establishing the role of climate/weather on the pathways that lead to human exposure. For example, in the air pollution–related health impact assessment, we investigated the relationships between weather and air pollution levels. In the vectorborne assessment, we focused on the relationships between climate and vector survival/activity and/or parasite development. These relationships were then applied using risk assessment methods to estimate the burden of disease under different scenarios. The sections that follow describe these relationships in more detail as well as how they were applied in the risk assessment process.

## Health Impacts in the Future: The Use of Scenarios

Estimating the potential impact of climate change on human health calls for the development of risk assessment methods based on scenarios that provide a description of how the future may develop on the basis of plausible and internally consistent sets of assumptions (Ebi and Gamble 2005).

Future climate scenarios for Portugal were created from two regional climate models (RCMs): PROMES, a regional climate model for the Iberian Peninsula developed at the Universidad Complutense de Madrid (Gallardo et al. 2001), and HadRM2, a regional climate model for Europe developed at the Hadley Centre (Exeter, UK), United Kingdom (Jones et al. 1997). Although both RCMs are nested inside the same global climate model (HadCM2) and have similar grid resolutions of approximately 50 km, they differ regarding time frames and future carbon dioxide (CO_2_) concentrations (Table 2). These differences result in two different sets of projected climate conditions. Because of the difference in the target decades and CO_2_ concentrations, results from the two RCMs cannot be directly compared. Nevertheless, the PROMES climate change model projects a less warm scenario than the HadRM2 model. The results of these models were used in this study to allow for a simple sensitivity assessment of how each health outcome could be affected.

Because both RCM control runs had CO_2_ concentrations similar to those observed during the end of the 20th century, control model runs from both RCMs were compared with (observed) climatology for the baseline period 1961–1990 (Miranda et al. 2002). These comparisons proved to be realistic, although they show that HadRM2 had better agreement with observations than did PROMES. As expected, differences between RCMs and observed climate were more noticeable when extreme weather conditions were compared than when examining average climate.

Compared with the control scenario, the climate change projections for both models show substantial increases in mean annual air temperature, with PROMES indicating an increase of approximately 3.3°C for the 2040s, and HadRM2 5.8°C by the 2090s. This warming is not uniform throughout the year or in its geographic distribution. HadRM2 comparisons project average minimum temperature increases during the winter of the order of 4.5–5.5°C, with greater increases in the interior south. Changes in summer average maximum temperature are projected to increase by 4.5–9.5°C, with the northern interior experiencing the maximum increase. Similarly, PROMES anomalies project increases in the winter average minimum temperature ranging from 3.1 to 3.3°C, with highest increases in the south. However, PROMES summer average maximum temperature anomalies project increases of 4–4.5°C, with the maximum anomaly in the southwest coast. Both models project increases in the number of days per year with maximum temperatures above 35°C, as well as days with minimum temperatures above 20°C. Increased frequency in the days with heavy daily precipitation events in winter are projected by both models. However, HadRM2 projects reductions in mean annual precipitation and in the duration of the rainy season, whereas PROMES projects mean precipitation increases.

Observed climate conditions were used to establish the climate–health relationships (heat-related and air pollution–related impact assessments) as well as in the assessment of current health impacts. Results from both control and future runs of RCMs were used to assess the potential changes of each health outcome.

For the heat-related mortality assessment, additional daily weather scenarios for maximum temperature were projected (using both RCMs) for the 2020s and 2050s. Analysis of the mean maximum temperature changes showed that PROMES predicts a slightly warmer climate than the HadRM2 for the 2020s and 2050s (Dessai 2003). These projections were not available for other climate variables, limiting their use to the heat-related mortality assessment.

The population/demographic projections developed by the SIAM socioeconomic group could not be used because they were not available at the city/district level. Thus, the population projections for Lisbon used in the heat-related mortality assessment were constructed (Dessai 2003) to be consistent with the Intergovernmental Panel on Climate Change Standardized Reference Emission Scenarios (Nakicenovic and Swart 2000). Lisbon’s population was projected to grow in all scenarios. The median population from these calculations for the respective time periods was used in the heat-related mortality assessment (Dessai 2003).

Ecologic scenarios were developed and used in the vectorborne disease assessment. These scenarios incorporate a range of assumptions about the vectors (see below).

## Heat-Related Mortality

Heat-related deaths occur during heat wave periods in Portugal (Garcia et al. 1999). An empirical-statistical model, developed and validated by Dessai (2002), was used to estimate future changes in heat-related mortality in Lisbon. The model used an exposure–response relationship derived from observed daily maximum temperatures and mortality during the summer months of 1980–1998. It was assumed that no adaptation occurred during this period.

The climate–mortality association was estimated using an observed–expected analysis similar to the method applied by Guest et al. (1999). Two approaches were used to calculate the number of deaths in excess of the number that would have been expected for that population (during the same period) in the absence of stressful weather. The first approach used a fixed mean of daily mortality for each summer month for the entire period 1980–1998. The second approach applied a 30-day running mean from mid-May to mid-September but selecting only the summer values (thus having a different value for each summer day). Changes in cold-related deaths were not assessed nor were the deaths resulting from higher air pollution levels in hotter weather.

Dessai (2002) observed that both approaches produced consistent results; heat-related deaths were not discernible below 29°C. A nonlinear regression method (of the type *y* = *ae^bx^*) showed a strong relationship between maximum temperature and excess deaths (for both approaches) in Lisbon. Using this relationship, under the fixed-summer-months mean approach (*a* = 0.00002 and *b* = 0.3744), total annual heat-related mortality in Lisbon was estimated at 6 per 100,000 (128 deaths per year) for the period 1980–1998. The second approach (*a* = 0.000006 and *b* = 0.4113) estimated an annual heat-related mortality of 5.4 per 100,000 for the same period. These results were used to estimate potential heat-related deaths in Lisbon under the population assumptions and climate change scenarios (Dessai 2003). These projections showed a consistent increase in death rates (Table 3), reaching mortality rates of 29.5 and 16.6 per 100,000 in the 2050s using PROMES and HadRM2, respectively. The difference between the two numbers is due to the use of not only different scenarios but also different methods of calculating the current annual heat-related mortality.

## Air Pollution–Related Health Effects

The adverse health effects associated with air pollutants such as nitrogen dioxide (NO_2_) and tropospheric ozone (O_3_) have been widely described. In this assessment we explored the possible trend in air pollution–related health effects based on expected meteorologic changes. We investigated the association between ambient levels of NO_2_ and O_3_ in Lisbon with temperature and wind speed. The climate–pollutant estimates were then used to determine potential changes in the levels of these pollutants in Lisbon under different climate scenarios. Potential changes in health outcomes due to these pollutant level changes were qualitatively assessed. Quantification of potential health outcomes was not possible because data on hospital emergency admissions or on daily mortality for the relevant period were not available.

Analysis of NO_2_ and O_3_ levels from the Lisbon monitoring air pollution network (DGA 2000) indicated that ambient air levels in Lisbon often exceed health-based air quality standards. NO_2_ ambient concentrations in the winter were significantly higher than in summer months. Previous studies of air quality in Lisbon found the highest NO_2_ pollution levels on cold days with wind speeds below 2 m/sec (Andrade 1996). In both scenarios future climate conditions will become less favorable for high ambient NO_2_ levels in Lisbon because the number of cold days with low wind speeds is expected to decrease (Table 4). Consequently, if current air pollution emission levels were maintained, NO_2_ levels in winter likely would decrease. Reductions in health burdens associated with acute ambient NO_2_ exposures, such as exacerbation of asthma, eye irritation, and respiratory tract infections, may occur, especially in winter.

Results from the same air quality monitoring network also indicated that ambient O_3_ levels in Lisbon are higher in the summer months. A direct correlation between temperature and O_3_ levels was observed. Studies from other cities confirm similar relationships and indicate that the simultaneous occurrence of daily maximum temperatures > 25°C and low wind speeds favor the occurrence of summer-time high O_3_ episodes (Anderson et al. 2001). Table 4 shows that in both scenarios future climate conditions will become more favorable for high ambient O_3_ levels in Lisbon, because the number of warm days with low wind speeds is expected to increase. Higher O_3_ concentrations may induce short-term reductions in lung functions within the “healthy population” and exacerbate current chronic respiratory diseases such as asthma, which presently pose significant public health concerns.

## Vectorborne Diseases

Mosquito-borne diseases such as malaria were a major public health concern in Portugal until the 1950s, and diseases transmitted by other vectors, such as Mediterranean spotted fever (MSF) and leishmaniasis, remain endemic to Portugal. Although human cases of vector-borne diseases have generally decreased over recent decades, many competent vectors are present in Portugal, posing a disease risk. Vectors and, indeed, some vectorborne diseases often exhibit distinct seasonal patterns that suggest that they are weather sensitive (Caeiro 1999; Pires 2000; Sousa et al. 2003). We assessed whether climate change may alter the risk levels of contracting vectorborne diseases in Portugal.

Diseases included in the assessment were identified based on published literature and consultations with national vector biologists and public health professionals; diseases considered in this article include malaria, West Nile virus (WNV) fever, leishmaniasis, Lyme disease, MSF, and schistosomiasis. Information on current and historical disease prevalence, vector presence, appropriate hosts, and parasite prevalence was compiled from official national records, an extensive literature review, and laboratory records. Temperature threshold limits for pathogen and vector survival were obtained from the literature, as summarized in Tables 1 and 5.

Disease transmission risk was categorized qualitatively based on vector abundance and pathogen prevalence (Table 6). Because there were knowledge gaps regarding the current presence, distribution, and abundance of vectors and pathogens, disease transmission risk levels were estimated for the various scenarios based on different assumptions of vector and parasite prevalence, together with climate scenarios (Table 7). In this study, vector survival/activity periods were used as indicators of vector abundance. It was assumed that longer pathogen survival periods would increase the number of organisms in the vectors. The lengths of these survival periods were estimated as the percentage of days per year that were categorized as having favorable temperature threshold limits.

The percentage of days per year with favorable temperature limits was calculated for each grid point and for each climate scenario using daily mean temperature values obtained from PROMES and HadRM2. The results were then grouped into the five administrative regions of Portugal, and the means for each region were estimated. Observed daily mean temperatures for key locations within each of these five regions were used to calculate the current percentage of days per year with favorable temperature limits in these regions. Table 8 shows the results for three of these administrative regions.

It is important to note that because the study approach relied heavily on temperature thresholds, the assessment gives only an indication of the temperature-induced change in transmission potential under several climate change scenarios. Although temperature is a key factor in disease transmission dynamics, other factors such as water-breeding sites, humidity, and wind also influence disease transmission. Moreover, disease transmission to humans requires human contact (exposure) with the parasite-infected vector. This exposure is influenced by a variety of factors, including human behavior, socioeconomic conditions, environmental management practices, and primary health care practices. Intrinsic factors such as immunity affect the severity of disease. Disease transmission occurs only if all factors are favorable for transmission.

### Malaria

The current Portuguese climate is conducive to malaria transmission, and competent vectors (*Anopheles atroparvus* mosquitoes) are abundant and widespread (Galão et al. 2002). The fact that no local malaria cases are reported indicates that the local mosquito population is not infected with parasites. Although survival of both vector and parasite is possible under current climate conditions (Tables 5 and 8), the current (Table 7, scenario 1) transmission risk of *Plasmodium vivax* malaria is very low (parasite and vector both present but no infected vector present), whereas that of *P. falciparum* malaria is negligible because no suitable vectors are present in Portugal. However, if a (new) population of mosquitoes infected with *P. vivax* or, alternatively, *P. falciparum* were to be introduced into Portugal and current environmental conditions assumed (scenario 2), transmission risk might change to a low risk level, assuming no additional vector control.

The climate change scenarios used projected significant increases in the number of days with mean temperatures suitable for *Anopheles*, *P. vivax*, and *P. falciparum* survival (Table 8). However, if no infected vectors are present (scenario 3), the risk of contracting *P. vivax* malaria should remain very low, and negligible for *P. falciparum* malaria. The risk might increase to a medium risk level if a new population of mosquitoes infected with *P. vivax* (or *P. falciparum*) were introduced (scenario 4). Higher risk levels are not anticipated because infected humans (hosts) would be treated for the disease.

### WNV fever

Currently, several mosquito species that are competent vectors of WNV are abundant and widespread in Portugal (Galão et al. 2002). The temperature thresholds for WNV survival are not documented, but laboratory studies indicate that the ability of competent vectors to transmit the virus is favored by higher temperatures (Dohm et al. 2002) and the vector’s temperature-dependent survival pattern. Vector survival and virus transmission dynamics were highest at 30°C (Mpho et al. 2002). In the present study, it was assumed that the temperature thresholds for WNV survival would be similar those for to vector survival.

Although no human cases have been recently reported in the Portuguese population, in the summer of 2004 two WNV cases linked to tourism in Portugal were reported in Ireland (Connell et al. 2004). Recent studies confirm WNV serologic reactivity (virus not isolated) in a few wild birds at certain locations (Formosinho et al. 2002), and four mosquitoes were positive for WNV (Almeida et al. 2004). Therefore, it is reasonable to conclude that the current risk (scenario 1) of contracting WNV is low. Focal introduction of a (new) population of infected mosquitoes would not change this risk level (scenario 2).

Climate change may lengthen survival periods of WNV-competent (*Anopheles*) mosquitoes (Table 8) and possibly allow infected hosts (birds) to change their geographic range. These could result in changes in virus prevalence rates and distribution. Therefore, climate change may increase WNV transmission risk (scenario 3) from low to a medium level.

### Leishmaniasis

Leishmaniasis is endemic in Portugal. Field studies confirm that the current environment is conducive to *Phlebotomus* sand-fly survival for several months and that *Leishmania* prevalence is relatively high in reservoir hosts (dogs) in several regions in Portugal (Pires 2000). The current (scenario 1) risk of leishmaniasis transmission is thus medium. Focal introductions of additional infected vectors (scenario 2) are not likely to change the disease risk level.

The data presented in Table 8 suggest that climate change might decrease the number of days suitable for *Phlebotomus ariasi* survival in all areas of Portugal except in the northern region. Because this sandfly vector currently predominates in the northern region (Pires 2000), national disease risk levels are not projected to change with changes in *Ph. ariasi* transmission dynamics in the remaining regions. Table 8 also indicates significant increases in days with favorable temperatures for *Ph. pernicious* (sandfly responsible for most infections in Portugal) activity for the whole of Portugal. Based on these projections, it seems reasonable to conclude that the risk of contracting leishmaniasis may become high (scenario 3). Introduction of additional infected sandflies (scenario 4) is not anticipated to change the risk level.

### Lyme disease

Lyme disease is an emerging disease in Portugal. *Ixodes ricinus* ticks are present throughout Portugal (Caeiro 1999), and are at times infected with *Borrelia lusitaniae* (de Michelis et al. 2000). Although *B. lusitaniae* has been considered to be non-pathogenic to humans, very recent evidence confirmed its pathogenic role in human cases in Portugal (Collares-Pereira et al. 2004).

In contrast to Northern Europe, the tick in the Iberian Peninsula is found throughout the year, and is more abundant during the cooler months (Caeiro 1999). This is to be expected because the tick is sensitive to prolonged heat and low soil moisture. Table 8 shows that climate change conditions will become less favorable for tick activity and hence disease transmission in southern Portugal, but more favorable in the central (not shown in Table 8) and northern regions. Because the human population in the southern regions is much smaller than in the rest of the country, and because these ticks are currently less abundant in the drier and warmer southern regions of Portugal, it is reasonable to conclude that the national prevalence rate of Lyme disease is not likely to decrease under future climatic conditions (scenario 3). In fact, it is anticipated that disease risk might increase as infected ticks and hosts widen their geographic distribution. Focal introduction of additional human pathogen-infected ticks is not anticipated to change transmission risk levels (scenarios 2 and 4).

### Mediterranean spotted fever

Portugal has a high incidence rate of MSF. Cases are reported throughout the year, with peaks during July, August, and September (Sousa et al. 2003) that coincide with the maximum activity period of the brown dog tick, *Rhipicephalus sanguineus* (Caeiro 1999). Field studies confirm the abundant and widespread distribution of the tick as well as the high prevalence of dogs infected with *Rickettsia conorii* (Bacellar et al. 1995). Current (scenario 1) disease transmission risk is obviously high and not likely to decrease with the introduction of more infected vectors (scenario 2).

Predicting changes in MSF is very difficult because there is no simple correlation between specific climate variables and vector or pathogen survival. However, because *R. sanguineus* has a remarkable ability to adapt to its environment, and disease transmission is highest during warmer months, even in harsher arid climatic zones where ambient temperatures exceed 35°C and soil temperatures exceed 45°C (Mumcuoglu et al. 1993), disease transmission risk levels are not expected to decrease for any of the scenarios investigated in the study. In fact, it is possible that climate change may prolong the peak season of MSF cases because of higher temperatures in spring and autumn.

### Schistosomiasis

Half a century ago, endemic *Schistosoma haematobium* infections were known to occur in the Algarve region (Grácio 1981). Currently, only imported cases are reported in Portugal. Although current environmental conditions remain conducive to *Schistosoma* transmission, the competent snail population is currently not infected, so the present (scenario 1) risk of transmission is very low. Assuming ambient air temperatures as approximations of shallow water temperatures (which affect parasite and vector survival), it is clear that climate change might lengthen parasite survival periods (Table 8) and vector survival. However, if the local snail population remains uninfected (scenario 3), transmission risk would remain very low.

Focal introduction of the parasite from infected imported human cases to the currently noninfected snail population is also possible. If a focal parasite-infected snail population were to occur, and current climatic conditions are assumed (scenario 2), the transmission risk for schistosomiasis would be low because of the focal vector distribution (Table 6) and the favorable temperatures for parasite survival are limited to about half a year (Table 8). However, if a warmer climate scenario is assumed (scenario 4), and that the infected vector population may with time widen its geographic distribution as the favorable temperature period for survival increases significantly (Table 8), then disease transmission risk may increase toward a medium level.

## Discussion and Conclusions

Few published studies describe changes in the burden of climate-sensitive diseases in Portugal in response to changes in weather and climate. This makes identification of the potential future health impacts of climate change difficult. The present assessment focused on three potential climate change–related health impacts: heat-related mortality, air pollution–related health effects, and vector-borne diseases. Because in this study the burden of climate-sensitive diseases was not quantified for all impacts assessed, based on the results presented here together with the fact that the urban population in Portugal is getting larger and older, heat-related mortality is likely to be of the highest public health concern.

The assessment results indicate that during 1980–1998, the mean annual heat-related mortality rate in Lisbon was between 5.4 and 6 deaths per 100,000 individuals. Earlier studies showed that in moderate regions, a decline in winter mortality could possibly counterbalance the mortality increase during summer. This study did not assess cold-related mortality in Lisbon, which has not been fully addressed by others. Cold-related mortality is very difficult to estimate because of the many (non-climate) factors that contribute to deaths during winter months. For example, a preliminary evaluation of winter mortality after the 2003 heat wave in Portugal showed an increase in expected (total) winter deaths, some of which were associated with an influenza outbreak that winter (de Andrade 2004).

The annual heat mortality rate was projected to increase under all climate change scenarios, reaching rates of between 16.2 and 29.5 per 100,000 individuals by the 2050s, assuming that no additional adaptations were implemented. This large range is due to the many uncertainties, including the differences of each RCM’s future climate conditions and the performance of the model outside its temperature limits (the heat–mortality model was not validated for temperatures above those observed in 1980–98).

Analysis of current ambient levels of NO_2_ and O_3_ in Lisbon showed that pollution levels often exceed health-based air quality standards. Warmer and more variable weather, as projected by PROMES and HadRM2, might result in better dispersion of NO_2_ in winter, thus reducing its ambient levels, whereas the increased temperatures may reduce air quality in warmer months by increasing O_3_ levels.

Table 9 summarizes the risk of transmission of the vectorborne diseases assessed. These results suggest that (regional/local) climate change may increase the risk levels of zoonoses, such as leishmaniasis, Lyme disease, and MSF, which currently pose the greatest risk to public health. Diseases that currently have lower transmission risk levels such as malaria and schistosomiasis are more sensitive to the introduction of infected vectors than to local temperature changes. The current risk of (local) introduction of a new population of infected vectors was not assessed but is influenced by factors ranging from global trade and population movements from countries where the disease is endemic, to alterations in the geographic range of infected vectors due to ecologic changes or vector control management practices. In general, climate change has the potential to change most of these factors to favor an increase (global) risk of infected vector introduction as well as imported (human) disease cases.

Although the results of the project may appear to be too distant in the future for immediate actions, the results have been useful in some national policies. For example, the results of the SIAM project were used in the formulation of the Portuguese Climate Change National Program (PNAC) as well as in the Third National Communication to the United Nations Framework Convention on Climate Change to identify potential impacts and adaptation measures. However, actions related to the PNAC have so far focused on issues surrounding reduction of greenhouse gas emissions. In addition, the Health Ministry’s National Contingency Plan for Heat Waves (PCOD), which was established after the dramatic impact of the 2003 heat wave on mortality in Portugal, also made use of these results and other (inter)national research projects to identify current and potential future threats and most appropriate measures for adaptation to the impacts of climate and weather on human health. As a direct consequence of the PCOD, Portugal has an operative national heat wave early warning system with the objective of reducing adverse health effects of future heat wave episodes. How this system can be improved and validated is currently on the national research agenda.

Overall, the assessment results based on the RCMs used in this study are consistent in the potential direction of change for each health outcome. The scarcity of health and environmental data and the significant number of knowledge gaps in the relationship between climate and health resulted in many uncertainties. Actions required and research gaps identified during the assessment include *a*) improved hospital emergency records so that the relationships between morbidity and exposures to ambient air pollutants as well as thermal extremes can be determined, and *b*) improved disease, vector, and pathogen monitoring and surveillance to better understand vectorborne disease transmission and the associations between disease transmission and climate in Portugal. These gaps need to be urgently addressed in order to conduct more profound national assessments on public health vulnerability to climate changes.

## Figures and Tables

**Table 1:** Climate sensitive health outcomes selected: reason for concerns and data available.

Health outcome	Reasons for concern	Data available
Heat-related mortality	1,906 excess daily deaths during 1981 heat wave in Portugal (Garcia et al. 1999) Increasing elderly population and urbanization	Daily mortality (all causes) for Lisbon during 1980–1998 (Instituto Nacional de Estatística 2000) Daily climate data set for Lisbon [Instituto de Meteorolgia (IM) 2000]
Air pollution–related health effects	Prevalence rate of childhood asthma is 10% and for rhinitis 27% (Allergonet 2000) Respiratory disorders contribute to 16% of all deaths [Direcção Geral da Saúde (DGS) 2001a] Air quality guidelines for NO_2_ and O_3_ are often exceeded in urban regions	Daily NO_2_ and O_3_ concentrations in Lisbon [Direcção Geral do Ambiente (DGA) 2000] Daily climate data set for Lisbon (IM 2000).
Vectorborne diseases Malaria	Disease endemic in the past; currently an annual average of 80 imported malaria cases are reported (incidence of 0.8 per 100,000) (DGS 2001b) Malaria-competent vector is widespread and abundant (Ribeiro et al. 1988)	Vector survival– and parasite developmental rate– temperature relationships (Martens 1998)
WNV fever	Virus isolated from competent mosquito in 1996 (Fernandes et al. 1998) Competent vectors are widespread and abundant (Ribeiro et al. 1988)	Vector survival–temperature relationships (Martens 1998)
Leishmaniasis	Endemic disease with annual average of 15 cases reported (incidence of 0.15/100,000) (DGS 2001b) Competent vectors present (Pires 2000) Reservoir hosts (dogs) with *Leishmania infantum* infection prevalence up to 11.4% (Campino et al. 1995)	Vector activity– and survival-temperature relationships (Rioux et al. 1985; Tesh et al.1992; Theodor 1936)
Lyme disease	Endemic disease with 20 cases hospitalized during 1994–1999 [Instituto de Gestão Informática e Financeira da Saúde (IGIF) 2000] Competent vector and suitable hosts present (Caeiro 1999)	Vector activity–temperature relationship (Caeiro 1992; Sonenshine 1993)
Mediterranean spotted fever	Endemic disease with annual average of 800–1,000 cases reported (incidence of 9.8 per 100,000) (DGS 2001b) Competent vector widespread and abundant (Caeiro 1999) Reservoir hosts (dogs) with infection prevalence up to 85.5% (Bacellar et al. 1995)	Monthly number of reported cases (DGS 2001b)
Schistosomiasis	Disease endemic in the past, currently an annual average of 35 imported cases hospitalized (IGIF 2000) Competent vector present (Grácio 1981)	Vector survival– and parasite development– temperature relationships (Martens 1998)

**Table 2:** RCM scenario comparisons.

Scenarios	PROMES	HadRM2
Time frame representing control climate conditions (control climate scenario)	1981–1990	2006–2036^a^
Time frame representing future climate conditions (future climate scenario)	2040–2049	2080–2100
CO_2_ concentration for control scenario (ppmv)	± 330	± 330
CO_2_ concentration for future scenario (ppmv)	470–500	610–705
Mean annual temperature (ºC) increase between control and future climate scenarios	~ 3.3°C	~ 5.8°C

ppmv, parts per million by volume.

a Control simulation was performed for various decades with a constant value of CO_2_ concentration comparable with climatology in the baseline period 1961–1990. The period available for use in this assessment was 2006–2036.

**Table 3:** Modeled mortality rates (per 100,000 individuals) in Lisbon using observed climate and regional model climate scenarios.

		Climate data used			
		2020s scenario		2050s scenario	
Model study approach	Observed (1980–1998)	PROMES	HadRM2	PROMES	HadRM2
Summer months fixed mean	6	12.1	9.1	28.8	16.6
30-day running mean	5.4	11.5	8.5	29.5	16.2

**Table 4:** Meteorologic conditions conducive to high NO_2_ and/or O_3_ levels in Lisbon.

		Percent change	
Meteorologic condition	Observed climate for 1990–1999 (average number of days/year)	PROMES	HadRM2
Wind speeds ≤ 2 m/sec^a^^,^^b^	30	−1	−1
Wind speeds ≤ 2 m/sec and *T*_min_ ≤ 0°C^a^	0	−0.3	−0.5
Wind speeds ≤ 2 m/sec and *T*_min_ ≤ 5°C^a^	2	−1.5	−1
Wind speeds ≤ 2 m/sec and *T*_min_ ≤ 10°C^a^	10	−2.5	−0.1
*T*_max_ ≥ 25°C^b^	100	26	12
*T*_max_ ≥ 25°C and wind speeds ≤ 2 m/sec^b^	9	0.5	0.1
*T*_max_ ≥ 25°C and wind speeds ≤ 2.5 m/sec^b^	10	2	1
*T*_max_ ≥ 25°C and wind speeds ≤ 3 m/sec^b^	21	4	2

Abbreviations: *T*_max_, maximum temperature; *T*_min_, minimum temperature.

a Conducive to high NO_2_.

b Conducive to high O_3_.

**Table 5:** Current knowledge on vectorborne diseases in Portugal and favorable temperature threshold limits used in the assessment.

Disease	Suitable vector	Parasite
Malaria (*vivax*)	*Anopheles atroparvus*	*Plasmodium vivax*^a^
	Survival temp: 10–40°C	Survival temp: 14.5–35°C
Malaria (*falciparum*)	No known competent vector present	*Plasmodium falciparum*^a^
		Survival temp: 16–35°C
WNV fever	*Anopheles atroparvus* (see above) and *Culex* spp.	West Nile virus
Leishmaniasis	*Phlebotomus perniciosus*	*Leishmania infantum*
	Activity temp: 15–28°C	
	*Phlebotomus ariasi*	
	Survival temp: 5–30°C	
Lyme disease	*Ixodes ricinus*	*Borrelia burgdorferi*
	Activity temp: 7–30°C	
MSF	*Rhipicephalus sanguineus*	*Rickettsia conorii*
Schistosomiasis (bilharzia)	*Bulinus* spp. and *Planorbarius metidjensis*	*Schistosoma hematobium*^a^
		Survival temp: 15–39°C

temp, temperature. Adapted from Casimiro and Calheiros (2002).

a Imported human cases only.

**Table 6:** Vectorborne disease risk-level criteria.

		Parasite		
Vector	None present	Imported human human cases only	Low prevalence in vectors/hosts	High prevalence in vectors/hosts
None present	Negligible risk	Negligible risk	Negligible risk	Negligible risk
Focal distribution	Negligible risk	Very low risk	Low risk	Low risk
Regional distribution	Negligible risk	Very low risk	Low risk	Medium risk
Widespread distribution	Negligible risk	Very low risk	Medium risk	High risk

**Table 7:** Scenarios used in vectorborne disease risk assessment.

Climate model scenario	Assuming current knowledge of vector and parasite prevalence in Portugal	Assuming the introduction of focal population of parasite-infected vectors into Portugal
Current climate	Scenario 1	Scenario 2
Climate change	Scenario 3	Scenario 4

**Table 8:** Periods favorable to vectorborne disease transmission based on HadRM2 and PROMES mean daily temperature results (control scenario) and observed climate.

	Percentage of days/year within favorable temperature range						
	Parasite survival			Vector survival		Vector activity	
Region in Portugal	*P. vivax*	*P. falciparum*	*Schistosoma*	*Anopheles*	*Ph. ariasi*	*Ph. perniciosus*	*I. ricinus*
Northern region							
PROMES	(37) 50	(30) 43	(34) 48	(60) 79	(91) 96	(34) 41	(81) 92
HadRM2	(28) 57	(23) 49	(26) 55	(55) 87	(90) 92	(26) 43	(79) 90
Observed (Porto)	45	37	40	75	90	39	88
Lisbon and Tagus Valley							
PROMES	(54) 75	(43) 62	(51) 71	(89) 99	(99) 92	(47) 58	(97) 92
HadRM2	(50) 86	(40) 75	(46) 84	(87) 99	(99) 91	(45) 69	(97) 91
Observed (Lisbon)	53	49	50	89	98	50	96
Algrave							
PROMES	(59) 81	(50) 68	(56) 77	(92) 99	(98) 89	(51) 60	(97) 89
HadRM2	(50) 86	(40) 75	(46) 83	(85) 99	(99) 89	(45) 65	(97) 89
Observed (Faro)	61	59	60	90	97	46	95

**Table 9:** Vectorborne disease transmission risks for Portugal.

	Transmission risk level			
Disease	Scenario 1	Scenario 2	Scenario 3	Scenario 4
Malaria				
*P. vivax*	Very low	Low	Very low	Low–medium
*P. falciparum*	Negligible	Low	Negligible	Low–medium
WNV fever	Low	Low	Low–medium	Low
Leishmaniasis	Medium	Medium	High	High
Lyme disease	Medium	Medium	Medium–high	Medium–high
MSF	High	High	High	High
Schistosomiasis	Very low	Low	Very low	Medium

Transmission risk levels are described in Table 6; scenarios are described in Table 7.
